# Notch pathway activation is essential for maintenance of stem-like cells in early tongue cancer

**DOI:** 10.18632/oncotarget.10419

**Published:** 2016-07-06

**Authors:** Pawan Upadhyay, Sudhir Nair, Ekjot Kaur, Jyotirmoi Aich, Prachi Dani, Vidyalakshmi Sethunath, Nilesh Gardi, Pratik Chandrani, Mukul Godbole, Kavita Sonawane, Ratnam Prasad, Sadhana Kannan, Beamon Agarwal, Shubhada Kane, Sudeep Gupta, Shilpee Dutt, Amit Dutt

**Affiliations:** ^1^ Integrated Genomics Laboratory, Advanced Centre for Treatment, Research and Education In Cancer, Tata Memorial Centre, Navi Mumbai- 410210, India; ^2^ Division of Head and Neck Oncology, Department of Surgical Oncology, Tata Memorial Hospital, Tata Memorial Centre,Mumbai- 4100012, India; ^3^ Shilpee Laboratory, Advanced Centre for Treatment, Research and Education In Cancer, Tata Memorial Centre, Navi Mumbai- 410210, India; ^4^ Advanced Centre for Treatment, Research and Education In Cancer, Tata Memorial Centre, Navi Mumbai- 410210, India; ^5^ Department of Pathology, Advanced Centre for Treatment, Research and Education In Cancer, Tata Memorial Centre, Navi Mumbai- 410210, India; ^6^ Department of Pathology, Tata Memorial Hospital, Tata Memorial Centre, Mumbai- 400012, India; ^7^ Department of Medical Oncology, Advanced Centre for Treatment, Research and Education In Cancer, Tata Memorial Centre, Mumbai- 400012, India

**Keywords:** early stage tongue cancer, exome and transcriptome sequencing, IHC based expression analysis, cancer stem cell-like feature, Notch pathway inhibitors

## Abstract

**Background:**

Notch pathway plays a complex role depending on cellular contexts: promotes stem cell maintenance or induces terminal differentiation in potential cancer-initiating cells; acts as an oncogene in lymphocytes and mammary tissue or plays a growth-suppressive role in leukemia, liver, skin, and head and neck cancer. Here, we present a novel clinical and functional significance of *NOTCH1* alterations in early stage tongue squamous cell carcinoma (TSCC).

**Patients and Methods:**

We analyzed the Notch signaling pathway in 68 early stage TSCC primary tumor samples by whole exome and transcriptome sequencing, real-time PCR based copy number, expression, immuno-histochemical, followed by cell based biochemical and functional assays.

**Results:**

We show, unlike TCGA HNSCC data set, *NOTCH1* harbors significantly lower frequency of inactivating mutations (4%); is somatically amplified; and, overexpressed in 31% and 37% of early stage TSCC patients, respectively. HNSCC cell lines over expressing *NOTCH1*, when plated in the absence of attachment, are enriched in stem cell markers and form spheroids. Furthermore, we show that inhibition of NOTCH activation by gamma secretase inhibitor or shRNA mediated knockdown of *NOTCH1* inhibits spheroid forming capacity, transformation, survival and migration of the HNSCC cells suggesting an oncogenic role of *NOTCH1* in TSCC. Clinically, Notch pathway activation is higher in tumors of non-smokers compared to smokers (50% Vs 18%, respectively, *P*=0.026) and is also associated with greater nodal positivity compared to its non-activation (93% Vs 64%, respectively, *P*=0.029).

**Conclusion:**

We anticipate that these results could form the basis for therapeutic targeting of NOTCH1 in tongue cancer.

## INTRODUCTION

Recent large-scale genome wide studies have underscored a complex role of *NOTCH1* as a candidate tumor suppressor harboring inactivating mutation and deletions, as well as a driver of tumorigenesis harboring activating missense mutations and amplifications in a context dependent manner in HNSCC, and other cancers [1-10]. In addition, Notch signaling pathway plays a significant role in the maintenance of cancer stem-like population of cells (CSCs) in several human cancers [11-15]. Inhibition of Notch signaling prevents the formation of secondary mammospheres by cell lines derived from primary breast cancer patient samples. However, the biological significance of cancer stem-like cells (CSCs) in HNSCC has not been well characterized.

To understand the role of Notch signaling pathway in early-stage (T1-T2) tongue tumors, we examined the mutational landscape, copy number alterations and differential expression of receptor, ligands, modifiers and target genes of the Notch pathway, along with effect of genetic and pharmacologic perturbation of Notch pathway on cancer stem-like cells (CSCs) features of HNSCC cells.

## RESULTS

All the samples with available genomic DNA were tested for the presence of HPV using MY09/11 PCR and E6 transcript PCR primers. 40 of 71 samples analyzed, all were found to be HPV negative. Where exome sequence was available, the absence of HPV was re-confirmed using HPVDetector, as previously described [16]. TSCC samples of Indian origin to be HPV negative is consistent with other studies [17-19].

### Notch pathway is activated in early TSCC patients

**Figure 1:**
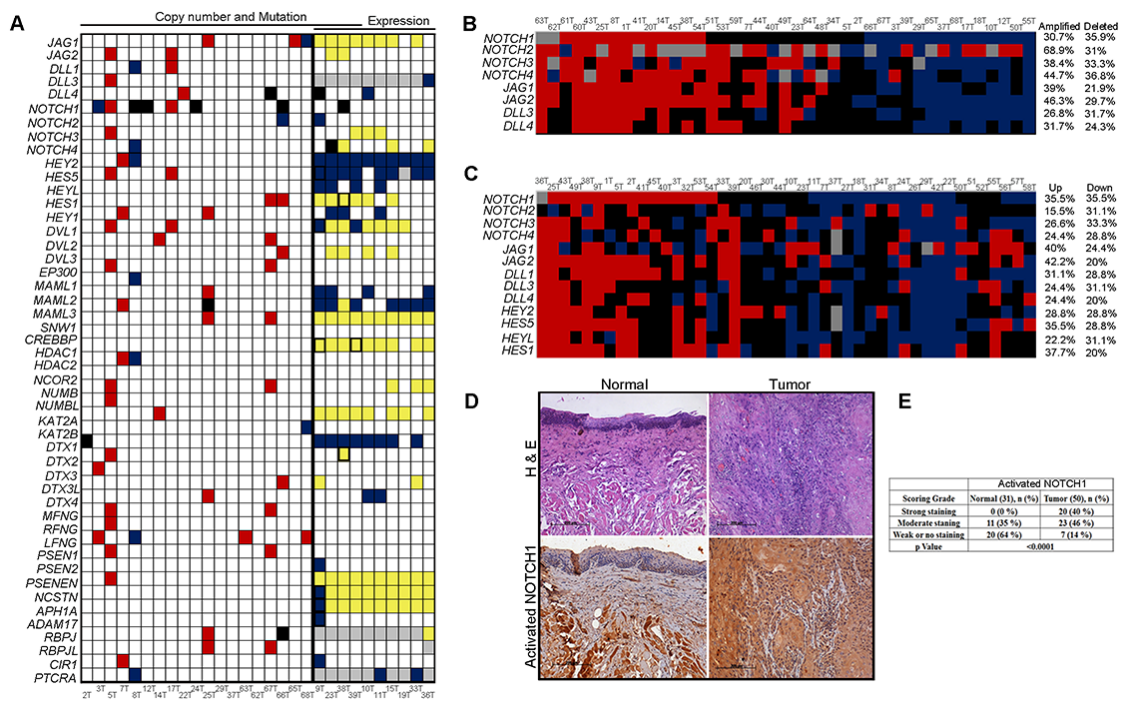
Activation of Notch pathway in early stage tongue squamous cell carcinoma **A.** Schematic representation of somatic mutation, copy number changes and expression changes identified in Notch pathway genes (*N*=48) using whole exome and transcriptome sequencing. Red filled; copy number gains, Yellow; high transcript expression, blue; copy number loss and low transcript expression, black; mutation, white; no events, grey; transcript not detected and black borderline boxes; any two events. Thick black line denoting separation of samples with exome and transcriptome sequencing. **B.** Schematic representation of DNA copy number alteration of Notch pathway genes in a cohort of 41 paired tumor samples estimated by quantitative real-time PCR. Red blocks; high copy number, blue; low copy number, black; diploid and grey color; experiment could not be done or data could not be acquired. **C.** Schematic representation of gene expression of Notch signaling pathway and its downstream targets in the cohort of 44 paired tongue tumor samples. Colors denotes: Red; upregulation, blue; down regulation, black; basal expression and grey color; experiment could not be done or results could not be acquired **D.** Immunohistochemistry (IHC) was performed for activated NOTCH1 in paired normal and tongue tumor samples (*N*=50). Brown color indicates positive expression. Representative IHC stained photomicrographs from normal and tongue tumor samples are shown. Scale bar, 100μM; corresponding H&E stained slides are shown in the upper panel. **E.** Tabular representation for quantification of activated NOTCH1 immunostaining data. Significant differences of IHC staining scores between normal and tumor were estimated using the Chi-square test and p value ≤0.05 was considered as threshold for statistical significance.

To characterize somatic alterations across 48 genes of Notch signaling pathway in 29 early-stage (T1-T2) tongue squamous cell carcinoma (TSCC) patient-derived tumors, we analyzed 23 paired whole exome and 10 whole transcriptome tongue cancer tumor sequencing data (unpublished data), as detailed in Supplementary Table S1 and Supplementary Figure S1A. Fourteen mutations were observed in 7 genes across 12 of 22 samples (Supplementary Table S1). Of note, inactivating *NOTCH1* mutation (4%) were found at a lower frequency in our sample set than that reported from the Caucasian population [7, 8, 20] but consistent with similar finding from a recent Asian study [21, 22]. In further contrast to Caucasian population, we observed Notch family receptors, ligands, and downstream effector genes were amplified or over expressed in 59% samples (17 of 29 patients) based on copy number variations called from whole- exome and whole- transcriptome data. To extend and validate these findings, we performed real-time quantitative PCR to estimate DNA copy number and transcript levels, along with an immunohistochemical analysis of Notch pathway components in paired tumor-normal samples from tongue cancer patients. We found somatic amplification at *NOTCH1* in 12 of 38 tumors (Figure 1A); overexpression of *NOTCH1* transcripts was observed in 16 of 45 samples (Figure 1B, 1C)– consistent with our analysis of the TCGA TSCC data set (n=126) (Supplementary Figure 1B, 1C), not reported earlier. Also, samples harboring amplification at *NOTCH1* (*P* value <0.001) and *DLL4* (*P* value <0.001) showed significantly higher expression of transcript as compared to no amplification. (Supplementary Figure S1D and Supplementary Figure S2). Consistent with amplification and over expression of Notch pathway components, Immunohistochemical analysis for activated NOTCH1 intracellular domain (NICD) in a set of 50 patients indicated strong immunoreactivity for active Notch signaling present in 40% tumor samples (Figure 1D-1E, Supplementary Figure S3A–S3C).

### Expression of *NOTCH1* is required for survival, migration and stemness of TSCC tumor cells

**Figure 2:**
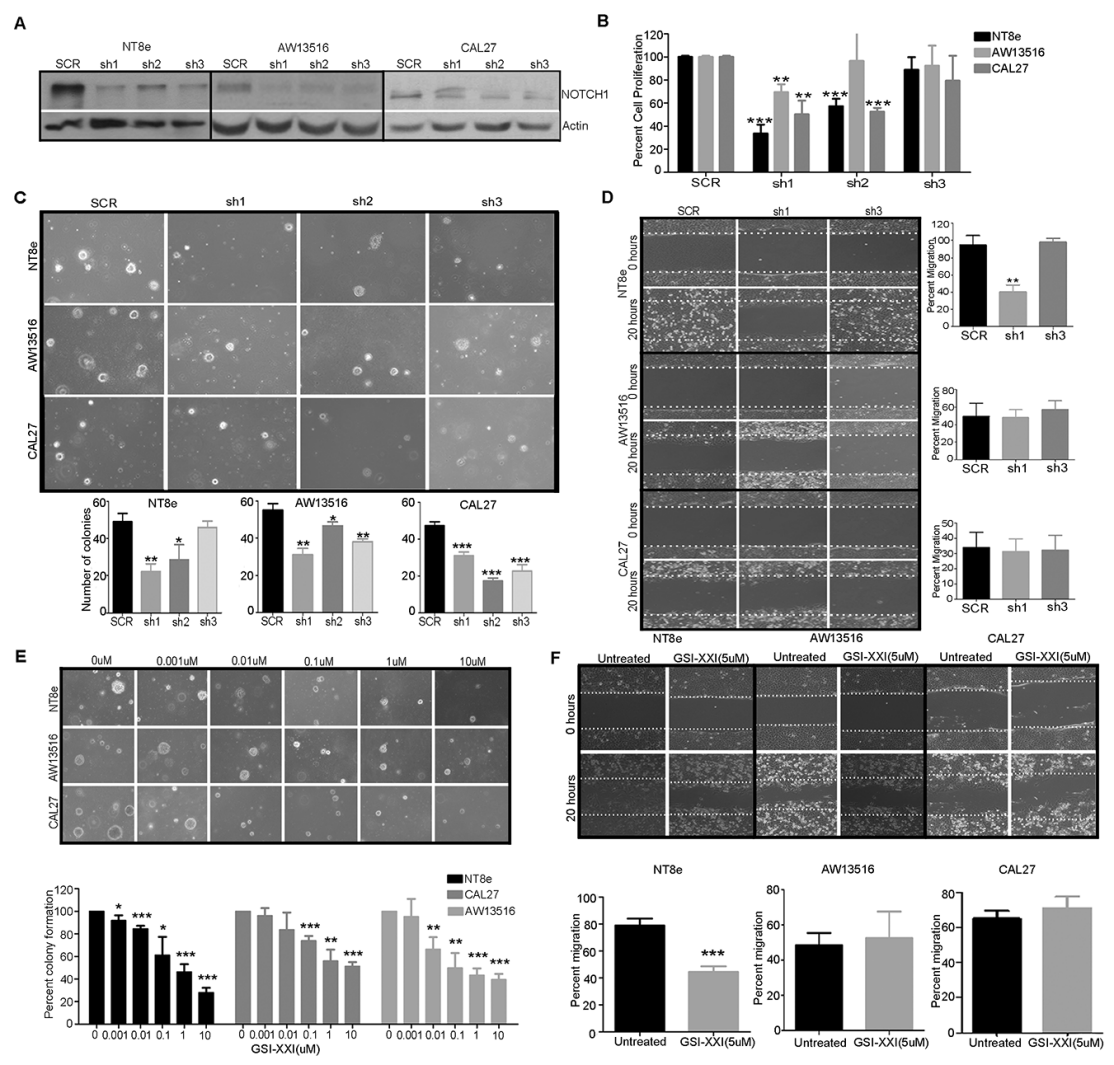
shRNA mediated knockdown and inhibition of NOTCH1 inhibits transformation, survival and migration of HNSCC cells **A.** shRNA constructs used to knock down *NOTCH1* expression in NT8e, AW13516, and CAL27 cells. Anti-NOTCH1 immunoblot shows that hairpins knock down to varying extents in different cells. Actin is included as a loading control. SCR, scrambled hairpin used as a negative control. **B.** Infection with 2 of 3 independent hairpins (sh*NOTCH1*#1 and sh*NOTCH1*#2) inhibited cell survival of NT8e and CAL27 cells expressing higher NOTCH1 levels as compared to the AW13516 cells– as assessed by plotting total cell count on day 6 compared to day 2, normalized against cells infected with SCR. **C.** Infection with independent hairpins inhibit soft agar colony formation by the NT8e and CAL27 cells expressing higher NOTCH1 levels compared to the AW13516 cells (upper panel). Colonies were photographed after 3 weeks (Magnification: ×10). Bar graph representation of soft agar colony formation (lower panel). **D.** Wound healing assay of knockdown clones of NT8e, CAL27 and AW13516 cells. NT8e cells with highest migration potential was most significantly inhibited following infection with sh*NOTCH1* constructs. Percent inhibition of migration was calculated after 20 hours of wound incision. **E.** Representative images of soft agar colony formation (upper panel) and bar graph representation of soft agar colony formation post gamma secretase inhibitor (GSI-XXI) treatment in HNSCC cell lines **F.** Wound healing assay of NT8e, CAL27 and AW13516 cells were performed post GSI-XXI inhibitor as indicated concentration and % migration was calculated after 20 hours of wound healing. Experiment was performed in triplicate and colonies were counted and shown as mean ± SD and P value is denoted as *; P < 0.01, **; P < 0.001, ***; P < 0.0001 versus non-targeting shRNA. Experiments were repeated two times independently.

To assess the functional significance of Notch pathway activation, we asked if the expression of *NOTCH1* is essential for survival, migration and stem-like feature of HNSCC cells *in vitro*. First, we checked for the presence of Notch pathway transcript expression by real-time PCR and western analysis of NOTCH1 protein using multiple head and neck cancers cell lines (NT8e, AW13516, CAL27 and DOK) [23]. NT8e and CAL27 cells showed higher expression of NOTCH1 as compared to AW13516 and DOK cells (Supplementary Figure S4A, S4B). Next, we tested a series of shRNA constructs to knockdown *NOTCH1* in these cells. The knockdowns were confirmed by western blot analysis for NOTCH1 (Figure 2A) and quantitative real-time PCR for *NOTCH1* and its target gene *HES1* (Supplementary Figure S4C). We identified two shRNA clones sh1 and sh2 that efficiently knocked down expression of *NOTCH1* compared to scrambled (SCR). Knock down of *NOTCH1* inhibited cell survival (Figure 2B), anchorage-independent growth (Figure 2C), in NT8e and CAL27, and migration in NT8e (Figure 2D).

**Figure 3:**
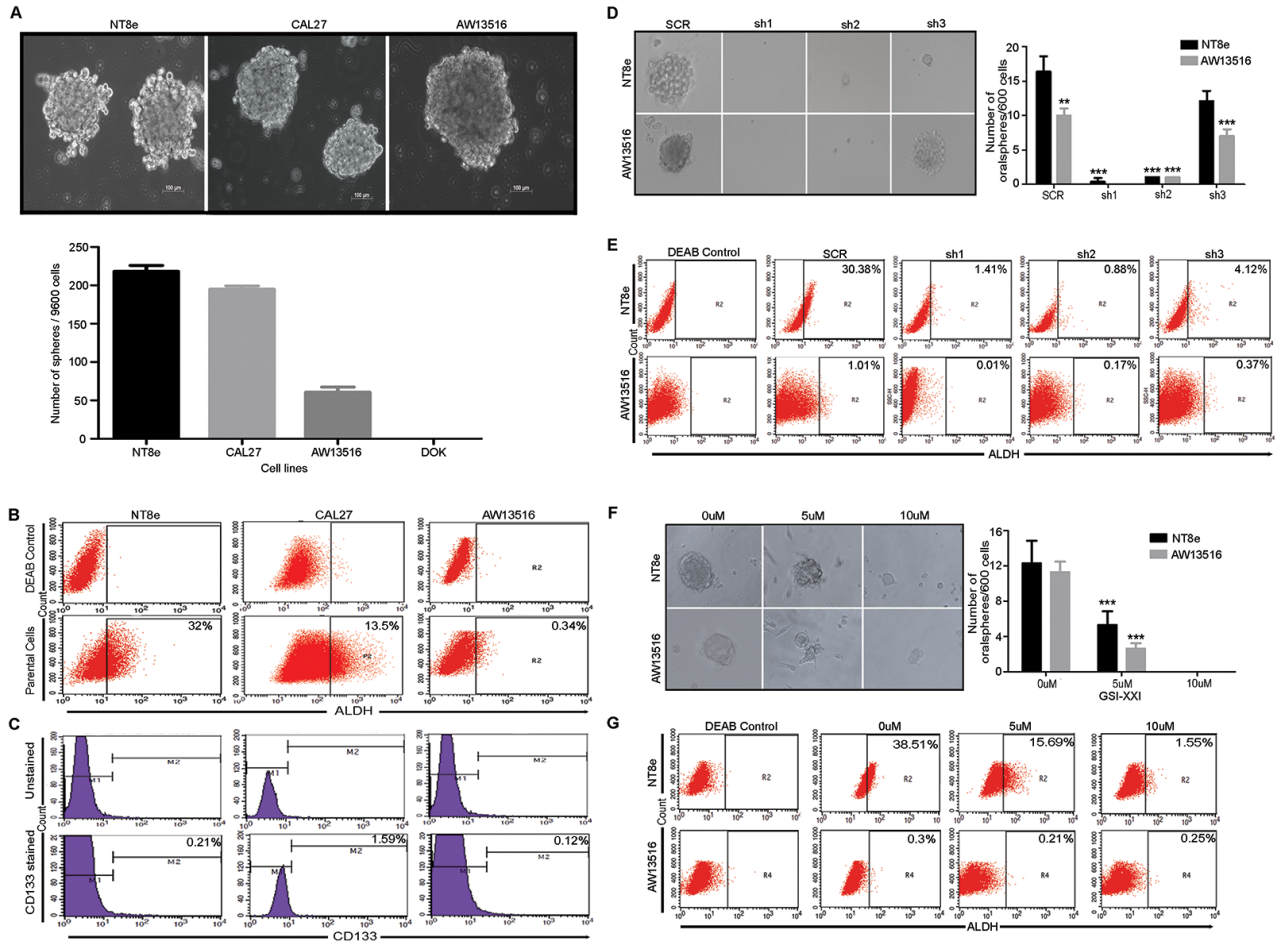
Notch pathway is essential for cancer stem-like property of HNSCC cells **A.** Oralsphere formation capacity of HNSCC cells. Representative images of oralsphere are shown in HNSCC cells. Oralsphere (>75μm size) were counted manually in triplicate via visualization under microscope and data was represented as Mean± SD. **B.** and **C.** Analysis of cancer stem-like cells (CSCs) marker ALDH and CD133 in HNSCC cells. Percentage ALDH positive cells were calculated against DEAB control. **D.** Representative images of oralsphere are shown in scrambled (SCR) and different shRNA clones (sh1, sh2 and sh3) of NT8e and AW13516 cells. Oralsphere formation assay was performed in triplicate and counting was done by observing under phase contrast microscope and data was represented as Mean± SD. **E.** ALDH staining for shRNA mediated knockdown clones and GSI-XXI treatment in HNSCC cells, respectively. Percentage ALDH positive cells were calculated against DEAB control. **F.** and **G.** Representative images of oralsphere post GSI-XXI treatment. NT8e and AW13516 cells post respective concentration treatment and ALDH positive cells. Number of oralsphere were counted and represented as Mean± SD. ALDH staining for shRNA mediated knockdown clones and GSI-XXI treatment in HNSCC cells, respectively. Percentage ALDH positive cells were calculated against DEAB control *P-value* *; ≥ 0.05 was considered as threshold for significance. All the above experiment were performed by at least two times independently by separate individuals.

Expression of *NOTCH1* and its pathway genes maintains cancer stem-like cells (CSCs) in various tumors, as determined by their ability to form spheroids and expression of molecular markers ALDH1, CD133 and CD44 [12, 24]. An *in vitro* spheroid formation assay was performed to examine the cancer stem cell population (CSCs) in HNSCC cell lines (NT8e, CAL27, AW13516, and DOK) expressing a variable level of *NOTCH1* expression (Supplementary Figure S4B). As shown in Figure 3A, following 10 days of incubation in undifferentiating stem cell media, NOTCH1 over expressing NT8e cells showed a higher number of oralspheres with 32% and 0.21% NT8e cells for cancer stem-like cells molecular marker such as ALDH and CD133, respectively. Similarly, *CAL27* cells also showed a significantly higher number of oralspheres with 13.5% and 1.59% CAL27 cells positive for ALDH and CD133 (Figure 3B, 3C). In contrast, AW13516 cells expressing comparatively lower NOTCH1 levels showed a reduced spheroid formation capacity with 0.34% ALDH positive and 0.12% CD133 positive while DOK cells did not show any oralsphere formation. To test whether a high fraction of the NT8e population constitutes the stem-like cells, we sorted NT8e cells in ALDH positive and ALDH negative fraction and assessed the sphere-forming efficiency (Supplementary Figure S5A). With subsequent passaging, the cells form ALDH negative population could not maintain their spheroid formation capacity while the ALDH positive population retained their self-renewal capacity demonstrating that indeed NT8e possess high ALDH positive cells are showing cancer stem-like cells features (Supplementary Figure S5B, S5C). *NOTCH1* knockdown clones showed significant reduction in oralsphere formation ability with concomitant decrease in ALDH positive cells in NT8e and AW13516 cells as compared to scrambled (SCR) cells (Figure 3D, 3E), highlighting their dependency on NOTCH1 expression with concomitant decrease in ALDH positive population of cells, thus regulating and promoting the survival of HNSCC CSCs. Next, we attempted to overexpress activated *NOTCH1* and full-length *NOTCH1* in AW13516 cells and checked for the sphere forming efficiency. Activated *NOTCH1* form more number of spheres as compared to vector control cells post 5 days (Supplementary Figure S6, S6C). However, given that AW13516 cells are HPV negative [25] and harbor wild-type *p16INK4A* and mutant *Tp53* [23], ectopic expression of full-length *NOTCH1* or *NICD* led to continuous cell death and senescence mediated growth arrest (Supplementary Figure S6, S6G), as described earlier [26-28].

### Notch pathway inhibitors block stem-like feature, proliferation, and survival of HNSCC cells over expressing NOTCH1

Finally, we investigated whether pharmacological inhibition of Notch pathway activation would be effective against HNSCC cell lines over expressing NOTCH1. Treatment of the NT8e, CAL27, AW13516 and DOK HNSCC cell lines with gamma secretase inhibitor (GSI-XXI) that abolished the presence of activated NOTCH1 (Supplementary Figure S4D) that resulted in significant reduction in soft agar colony formation (Figure 2E) and cell survival (Supplementary Figure S4E) as compared to vehicle treated in NT8e and CAL27 cells but not AW13516 cells. Additionally, the migration potential of NT8e cells was significantly inhibited by GSI-XXI, consistent with our observation using shRNA knockdown based approach (Figure 2F). Similarly, marked decrease in spheroid forming ability and ALDH expression in NT8e and AW13516 cells was observed post GSI-XXI treatment (Figure 3F, 3G).

### Activation of notch pathway correlates with node positive and non-smoker TSCC patients

Of particular significance is the correlation between clinicopathological characteristics and overall Notch pathway activation: immuno-histochemical based expression of activated NOTCH1 intracellular domain NICD (χ^2^=7.10, *P*=0.029), amplification at *DLL4* (χ^2^=7.5, *P*=0.023), and transcript over expression of Notch pathway effector genes *HEY2* (χ^2^=9.8, *P*=0.007) and *HES5* (χ^2^=5.71 *P*=0.057) significantly correlated with lymph node metastases (Table 1) and poor prognosis (Supplementary Figure S7A). Interestingly, this was consistent also with our analysis of the TCGA tongue cancer patient dataset (Supplementary Figure S7B), and with other cancers [24, 29, 30].

Table 1Clinical correlation analysis of Notch pathway alterations

**Table:** 

Clinicopathologic features	Variable	N (% along column)	*DLL3 Copy Number* (N=26) N (% along row)			P- value	N, % along column	*HEY2 Expression* (N=34) N (% along row)			P- value
			Gain (N=8)	Diploid (N=16)	Deleted (N=2)			Up (N=15)	Basal (N=12)	Down (N=7)	
Nodal Status	Node positive	19 (73%)	5 (26%)	14 (74%)	0 (0%)	**0.023**	22 (65%)	12 (55%)	9 (41%)	1 (5%)	**0.007**
	Node negative	7 (27%)	3 (43%)	2 (29%)	2 (29%)		12 (35%)	3 (25%)	3 (25%)	6 (50%)

**Table:** 

Clinicopathologic features	Variable	N (% along column)	*HES5* Expression (N=34) N (% along row)			P- value	N, % along column	Activated NOTCH1 IHC (N=49) N (% along row)			P- value
			Up (N=15)	Basal (N=12)	Down (N=7)			Strong (N=15)	Moderate (N=12)	Weak or No (N=7)	
Nodal Status	Node positive	23 (66%)	9 (39%)	12 (52%)	2 (9%)	0.057+	23 (66%)	13 (43%)	15 (50%)	2 (7%)	**0.029**
	Node negative	12 (34%)	4 (33%)	3 (25%)	5 (42%)		12 (34%)	6 (32%)	6 (32%)	7 (37%)

**Table:** 

Clinicopathologic features	Variable	N (% along column)	*NOTCH1* transcript expression (N=35), % along row			P- value
			Up (N=14)	Basal (N=9)	Down (N=12)	
Smoking	Smoker	11 (32%)	2 (18%)	6 (55%)	3 (27%)	**0.026**
	Non-smoker	24 (68%)	12 (50%)	3 (13%)	9 (38%)	

All clinical correlation analysis were performed in SPSS and significant alterations has been presented in the table. Patient's samples showing strong and moderate staining of activated NOTCH1 was considered as having activated Notch signaling. N; Number of samples, Up; upregulation, Down; downregulation. Chi-square test was used to calculate statistical significance. Significant *P-value* are highlighted in bold font. *P-value* ≤ 0.05 was considered as threshold for significance. + denotes; marginally significant.

In addition, *NOTCH1* expression significantly correlated with a non-smoking habit of patients (χ^2^=7.325, *P*=0.026), where 12 of 24 non-smokers patients derived tumors showed upregulation of *NOTCH1* transcript, consistent with previously described *NOTCH1* upregulation in non-smokers in other diseases including lung adenocarcinoma [31-33]. We also observed a significant correlation with AJCC (American Joint committee on Cancer) TNM tumor staging wherein stage III-IVA showed increases activation of NOTCH pathway (χ^2^=7.84, P=0.02). However, no statistically significant correlation was observed between the activated NOTCH1 expression with the sex, age, alcohol and tobacco consumption in the cohort, as represented in Supplementary Table S2. Next, we performed an interim analysis and assessed disease-free survival (DFS) by IHC defined activated NOTCH1 (strong and moderate staining) status vs non-activated NOTCH1 (weak or no staining) status. DFS was defined as time interval between the date of registration and the date of first documented evidence of relapse at any site (local, regional, metastatic, or secondary primary) or death from any cause, whichever earlier. There was no statistically significant difference was observed in tumors with activated NOTCH1 compared to those with non-activated NOTCH1 tumors, as shown in Supplementary Figure S7C.

Taken together, we present a novel clinicopathological correlation such that expression of Notch pathway components and activated NOTCH1 levels predispose TSCC patients to lymph node metastasis, and that non-smokers TSCC patients tend to have higher *NOTCH1* levels as compared to smokers. Clinically, determination of NICD by immuno-histochemistry could be a good predictor of nodal status. This could be a biomarker to predict lymph node metastasis for therapeutic utility among early stage tongue cancer patient to help patient stratification for treatment [34, 35].

## DISCUSSION

We demonstrate that 40% TSCC tumors have strong Notch pathway activation and that this property may be important in the maintenance of stem cell component in these tumors. Genetic or chemical perturbation of NOTCH pathway using shRNA and GSI-XXI showed decrease soft agar colony formation, migration potential and cancer stem-like features of HNSCC cells, highlighting their dependency on *NOTCH1* expression. Thus, targeted elimination of these cells may provide a new lead in treatment of head and neck cancer. These findings are consistent with reports where Notch signaling has been shown to be required for stem cell-like features in several cancer types [4, 13, 37]. Interestingly, genetic determinants of cancer stem cells share features with their role in the development of tumorigenesis [38]. These findings are consistent with reports where Notch signaling has been shown to be required for stem cell-like features in several cancer types, including HNSCC [14, 15].

Clinically, NOTCH1 transcript expression significantly correlate with non-smoking habit of patients, consistent with previous reports in other pathological conditions including lung adenocarcinoma [31-33]; lymph node metastasis in tongue cancer correlate with poor prognosis and survival of the patients, thus activated NOTCH1 could serve as a reliable marker to predict lymph node metastasis [35]. Moreover, AJCC TNM tumor stage III-IVA significantly correlates with activation of NOTCH pathway as compared to stage I-II, consistent with reports in HNSCC [39]. The sample size in this study, however, is underpowered to reach the statistical significance for survival data. No significant difference was observed in disease-free survival of the patients with IHC defined activated NOTCH1 tumors as compared to non-activated NOTCH1, as shown in Supplementary Figure S7C.

In conclusion, we demonstrate that a considerable fraction of TSCC tumors has upregulated Notch pathway and that this property may be important for the maintenance of stem cell component in these tumors. And that, NOTCH1 could be a potential therapeutic target in these patients.

## MATERIALS AND METHODS

### Patient Samples

Tumor-normal paired samples were collected at Tata Memorial Hospital and Advanced Centre for Treatment, Research and Education in Cancer (ACTREC), Mumbai. Sample set and study protocols were approved by (ACTREC-TMC) Internal Review Board (IRB) and most of the patients were recruited from 2010-2013 with a predefined inclusion criteria of early (pT1 and pT2) stage. Percent tumor content was determined using hematoxylin and eosin based staining by two independent pathologists which varied from 60 to 90%. Patient samples and characteristics are provided in the Supplementary Table S3.

### DNA and RNA extraction

DNA from tongue primary paired normal-tumor tissue samples were extracted using DNeasy Blood and tissue DNA extraction kit (Qiagen) according to manufacturer's instructions. DNA was quantified using Nanodrop 2000c Spectrophotometer (Thermo Fisher Scientific Inc.) and DNA quality was checked by resolving on 0.8% agarose gel. Total RNA was extracted from tongue primary paired normal-tumor samples and cell lines using RNeasy RNA isolation kit (Qiagen) and Trizol reagent (Invitrogen) based methods and later resolved on 1.2% Agarose gel to confirm the RNA integrity.

### Exome capture and NGS DNA sequencing

Two different Exome capture kits were used to capture exome for different samples. The TruSeq Exome Enrichment kit (Illumina) was used to capture 62Mb region of human genome comprising of 201,121 exons representing 20,974 gene sequences, including 5′UTR, 3′UTR, microRNAs and other non-coding RNA and NimbleGen SeqCap EZ Exome Library v3.0 was also used to capture 64 Mb region of the human genome. Exome library preparation and sequencing was performed as per manufacturer's instructions. Briefly, 2 μg genomic DNA was sheared using Covaris (Covaris Inc) for generating the fragment size of 200-300bp size. DNA libraries were prepared from both the kits were quantified by qPCR using KAPA Library Quant Kit (Kapa Biosystems) in ABI 7,900HT system (Life Technologies). Seven pmol of 6-plex DNA library pool was loaded per lane on flow cell (Flow Cell v3) to generate clusters using TruSeq PE (Paired-End) Cluster Kit v3-cBot-HS kit and clustered flow was sequenced for 201 cycles on HiSeq-1,500 System (Illumina) using TruSeq SBS Kit v3 (Illumina) at in-house core NGS facility.

### Identification of somatic mutations from exome Sequencing

Paired-end raw sequence reads generated were mapped to the human reference genome (build hg19) using BWA v. 0.6.2 [40]. Mapped reads were then used to identify and remove PCR duplicates using Picard tools v1.100 (http:broadinstitute.github.io/picard/). Base quality score recalibration and indel re-alignment performed and variants were called from each sample separately using GATK 2.5-2 Unified Genotyper and MuTect v. 1.0.27783 [41]. Post subtraction of variants from its paired normal, remaining variants was taken for further analysis if they were having ≥5 altered reads. Furthermore, all samples variants were further filtered against pooled normal variants database (N=62) to reduce the possibility of the germline variation We further annotated variants using Oncotator v1.1.6.0 [42] and dbSNP v142 [43] and COSMIC database v68 [44] using an in-house developed script. Later, we performed functional prediction tool based analysis for somatic non-synonymous variants using nine different tools such as: dbNSFP v2.0 (includes SIFT, Polyphen2_HDIV, Polyphen2_HVAR, LRT, Mutation Taster, Mutation Accessor and FATHMM) [45], CanDRA v1.0 [46] and Provean v.1.1 [47]. Variants called deleterious in nature by at least one software was taken for further analysis. We confirmed the identity of mutations by manual visualization in IGV [48, 49].

### Somatic copy number analysis from exome sequencing data

BAM files prepared for variant calling were used for copy number analysis using Control-FREEC [50]. Paired tumor-normal samples BAM files were fed into Control-FREEC along with target region for Illumina and Nimblegen exome kits as bed file. Read count were generated and normalized for GC content for each of the target region followed by computation of ratio of read count in a tumor to normal. Read count ratio was converted to copy numbers followed by segmentation using lasso method. Segmented copy number data generated by control-FREEC was further used for annotation and post-processing using R programming.

### Transcriptome sequencing and data analysis

Transcriptome libraries for sequencing were constructed according to the TruSeq RNA library protocol (Illumina). Briefly, mRNA was purified from 4 μg of intact total RNA using oligodT beads and library preparation was done as per manufacturer's instructions (TruSeq RNA Sample Preparation Kit, Illumina). 7pmol of quantified cDNA libraries were loaded on Illumina flow cell (v3) to generate clusters using TruSeq PE (Paired-End) Cluster Kit v3-cBot-HS kit and clustered flow was sequenced for 201 cycles on HiSeq-1500 System (Illumina) using TruSeq PE Cluster Kit v3 and TruSeq SBS Kit v3 (Illumina) to generate at least 30 million reads per sample. Post sequencing, de-multiplexing was carried out on the basis of index sequences using CASAVA (version 1.8.4, Illumina). Transcriptome data analysis was performed using Tuxedo-suite pipeline [51]. In brief, alignment of short reads was done against reference genome (hg19) using TOPHAT2 v. 2.0.8b [52] in which 95-99% of reads were mapped to the reference genome. Cufflinks v.2.0.2 was used to find the expressed transcripts in the data and quality control steps was performed using CummeRbund package v2.0. All the actively expressed transcripts per samples were then binned by log_10_(FPKM+1) to differentiate the significantly expressed transcripts from the background noise and transcripts represented by <0.1 log_10_(FPKM+1) were filtered out from further analysis. Since paired normal of these tumors cannot be obtained, we defined a significant change in expression for those genes whose expression is higher (>80%) or lower (<20%) than the median expression as suggested [53].

### Analysis of the cancer genome atlas tongue cancer data

The Cancer Genome Atlas (TCGA) dataset of HNSCC including DNA copy number dataset (gistic2 threshold) from 452 HNSCC tumor tissue, RNA seq expression (Illumina HiSeq) dataset from 541 HNSCC was downloaded from UCSC Cancer genome browser on 20^th^ June 2014. Later, tongue cancer patient data for DNA copy number (n=126) and gene expression (n=129 has been taken for further analysis. For expression and DNA copy number the median centered RSEM counts and gene-level copy number estimates have been used, respectively (n=126). Notch pathway genes (n=13) data has been retrieved and heatmaps were generated using MeV 4.9.0. The RNAseq gene expression data has been retrieved for Notch pathway genes (n=13) and raw data has been median centered using Cluster 3.0 software. The median centered RSEM counts for each gene has been used to generate heatmap using MeV 4.9.0. Fold change criteria was ≥1.5 fold change for upregulation, ≤1.499 fold change for down-regulation and in between −1.5 to 1.499 fold change was denoted as a basal expression. DNA copy number and Expression correlation analysis and clinical correlation analysis have been performed using SPSS. P=value <0.05 was criteria for statistical significance.

### Tissue processing

Surgically resected oral tumor tissues and matched nonmalignant (cut margins) adjacent tissues were obtained from patients with informed consent after IRB approval from ACTREC. These tissues were processed for paraffin embedding and sectioned at 4μm for H/E staining for evaluation of tumor.

### Immunohistochemistry

Immunohistochemistry was done following the standard protocol of DAKO Envision Flex. Briefly, the slides were microwaved by incubating them for 10 minutes in high pH antigen Retrieval Solution (DAKO;DM828), then allowed to cool to room temperature before rinsing with Tris-buffered saline wash buffer (DAKO;DM831). Endogenous peroxidase activity was blocked by incubating the slides for 20 minutes in 3% hydrogen peroxide (EnVision/HRP, Dako). After rinsing in wash buffer, the sections were incubated for 3hours at room temperature with the monoclonal human anti-activated Notch1 antibody (Cat.ab8925; dilution 1:50) in Tris-HCl buffer antibody diluent (Dako; K8016). Slides were rinsed in wash buffer (DAKO; DM831) and incubated for 90 minutes with peroxidase-labeled polymer conjugated to goat anti-rabbit immunoglobulins (EnVision/HRP, Dako; SM801). The chromogenic reaction was carried out with 3,3′-diaminobenzidine chromogen solution for 5 minutes, resulting in the expected brown-colored signal. Finally, after rinsing with deionized water, the slides were counterstained with hematoxylin, dehydrated, mounted with toluene-based mounting medium (Thermo Scientific Richard-Allan) and cover slip.

### Immunohistochemical staining analysis

Evaluation of immunohistochemical staining of activated Notch1 expression was scored as 0, 1+, 2+ and 3+. The percentage of cells with positive staining was scored from 0 to 4 (0=0% positive cells; 1: <10% positive cells; 2: 10-49% positive cells; 3: 50-80% positive cells; 4: >80% positive cells) and staining intensity was scored from 0 to 3 (0, negative; 1, weak; 2, moderate; 3, strong). The two scores were then multiplied. Final scores of 0-2 were scored as 0, 3-5 as 1, 6-8 as 2 and 9-12 as 3.6

### Quantitative real-time PCR for copy number analysis

Primers details used for copy number study has been provided in Supplementary Table S4. All primers used have been tested for their specificity by performing evaluative PCR as well as melt curve analysis during quantitative real-time PCR. Amplification efficiency for all primer was tested with series of dilutions (0.625 ng, 1.25 ng, 2.5 ng, 5 ng, 10 ng) of genomic DNA and PCR amplification efficiency was ~97%(~R^2^=0.979) (Supplementary Figure S8A, S8B). Based on above quality control, 10 ng of genomic DNA per 10 μl reaction volume in triplicates were run on Light cycler 480 (Roche, Mannheim, Germany) twice independently and relative copy number analysis was performed as described previously [54]. The threshold for calling high and low copy number was ≥2.5 and ≤1.5, respectively and ≤2.5 and 1.5≤; diploid.

### Quantitative real-time RT-PCR for expression analysis

Prepared cDNA was diluted 1:10 and reaction were performed in 10μl volume in triplicate. The melt curve analysis was performed to check the primer dimer or non-specific amplifications. Real-time PCR was carried out using KAPA master mix (KAPA SYBR® FAST Universal qPCR kit) in 10 μl volume in triplicate on Light cycler 480 (Roche, Mannheim, Germany) machine. All the experiments were repeated at least twice independently. The data was normalized with internal reference *GAPDH*, and analyzed by using delta-delta Ct method described previously The criteria were ≥2 fold change for upregulation, ≤0.5 fold change for down-regulation and in between 1.99-0.501 fold change as a basal expression. The details of all the primers used for expression analysis have been provided in Supplementary table S4.

### Cell culture

Cell lines established from different sub-sites of head and neck cancer: AW13516 from tongue, NT8e from upper aero-digestive tract, CAL27 cells from tongue [55] and partially transformed cell line DOK (tongue) [56] were used in this study. AW13516 and NT8e were acquired from Tata Memorial Hospital while CAL27 and DOK cells were procured from ATCC and Sigma, respectively. All cells were grown in Dulbecco's Modified Eagle Medium (Pan biotech, Germany). Culture media was supplemented with 10% FBS (Gibco, US), 1% Penicillin-Streptomycin solution (Sigma) and maintained at 37°C in an incubator with 5% CO2. DOK cells were grown with 5ug/ml hydrocortisone (Sigma) as a supplement. Trypsinization was performed using 0.25% Trypsin-EDTA (Invitrogen) and freezing of cells performed in 90 % FBS (Gibco, US) and 10% DMSO (Sigma) and were stored in liquid Nitrogen for long term storage. All the cell lines were authenticated using a short tandem repeat (STR) analysis kit (Gene Print v10, Promega, USA). The results are shown in Supplementary Table S5.

### Retrovirus production, infection and drug selection

Retroviral shRNA constructs were purchased from TransOMIC technologies, USA. Target sequences of *NOTCH1* shRNA constructs: sh1 5′-CAGTGAGCGATGACTGCACAGAGAGCTCCTAT-3′, sh2 5′-CAGTGAGCGATGGACGGACCCAACACTTACAT-3′, and sh3 5′-CAGTGAGCGAGACGAGGACCTGGAGACCAAAT-3′. 293T cells were seeded in 6 well plates one day before transfection and each construct (pMLP Retroviral-puro) along with pCL-ECO and pVSVG helper vector were transfected using Lipofectamine LTX reagent (Invitrogen). The viral soup was collected 48 and 72 hours post transfection, passed through 0.45μM filter and stored at 4OC. Respective cells for transduction were seeded one day before infection in a six-well plate and allowed to grow to reach 50-60% confluency. One ml of the virus soup (1:5 dilution) and 8μg/ml of polybrene (Sigma) was added to cells and incubated for six hours. Cells were maintained under puromycin (Sigma) selection.

### Overexpression of NOTCH1 and selection

The human full-length *NOTCH1* (pcDNA-*NOTCH1*) [57] was obtained from Artavanis-Tsakonas Laboratory (Havard Medical School) and activated *NOTCH1* (pEGFP-NICD) [58] constructs was obtained from Annapoorni Rangarajan (Indian Institute of Sciences (IISc), Bangalore, India). Cells expressing pcDNA-*NOTCH1* or pEGFP-NICD were generated by transfection with 10μg of DNA using Lipofectamine 3000 (Invitrogen) as per manufacturer's instructions. After 48hours, cells were cultures for 8-10 days in complete medium supplemented with 1mg/ml of G418 for antibiotics selection of transfected cells or cells were sorted based on GFP expression using BD FACSAria II. Pooled GFP sorted or antibiotics selected cells were later used for oralsphere assay. In case of 293T cells, post 48hours transfection cells were taken for RNA extraction and protein extraction for quantitative real-time PCR and western blot analysis, respectively.

### Western blotting

Cells were lysed in RIPA buffer (Sigma) and protein concentration was estimated using BCA (MP biomedicals) method [59]. Forty microgram protein was separated on 10% SDS-PAGE gel, transferred to nitrocellulose membrane and transfer was verified using Ponceau S (Sigma). Later the blots were blocked in Tris-buffered saline containing 5% BSA (Sigma) and 0.01% Tween-20(Sigma) and were probed with full-length NOTCH1 (sc-6014-R, Santacruz biotechnology), anti-activated NOTCH1 antibody (Abcam; ab8925) and anti-actin (A5316, Sigma) antibody. The membranes were then incubated with corresponding secondary HRP-conjugated antibodies (Santa Cruz Biotechnology, USA) and the immune complexes were visualized by Pierce ECL (Thermo Scientific, USA) according to manufacturer's protocol. Western blot experiments were performed in triplicate.

### Anchorage-independent growth assay

For analysis of growth in soft agar, 5 × 103 cells were seeded in triplicate onto a six-well dish (Falcon) in 4 ml of complete medium containing 0.33% agar solution along with respective treatments of GSI-XXI at 37°C in CO2 incubator. Ten images per well were photographed after 21 days using inverted phase contrast microscope and colonies were counted manually.

### MTT assay

A Thousand cells per well (six replicate per concentration) were seeded in 96-well plate followed by incubation with the drug for 72 hours and subsequently incubated with MTT (0.5 mg/ml) for 4 hours. Later, MTT assay was performed and data was acquired at 570nm using Microplate reader. Percentage cell viability was calculated against vehicle treated control.

### Wound healing assay

The cells were grown in 6 well plates to 95% confluency and were replaced with fresh medium containing 5/ml mitomycin C (Sigma). After 2 hours incubation, the medium was discarded and wounds were scratched with the help of sterile 10μl pipette tip. Cells were washed with PBS to remove the detached cells during creating a wound. The cells were fed with fresh medium and observed by time-lapse microscopy, and images were taken every 10 min for 20 hr. Migration was measured using Image J software.

### Oralsphere formation assay

Ninety-six hundred cells were seeded in 1.2 % agar coated 6-well plates supplemented with stem cell media (recombinant EGF (20 ng/ml), human basic FGF (20 ng/ml), L-glutamine (2 mM), B-27 supplement and N2 supplement) and allowed to grow for 10 days. After every five days media, additional media was supplemented. Five hundred cells from NT8e, AW13516 and CAL27 shRNA clones were seeded on an ultra-low adherent 96-well plate in stem cell medium. Oralspheres were then cultured and maintained in low adherent 24-well plates. Additionally, the parent NT8e, AW13516, and CAL27 cells were also checked for the spheroid formation capacity upon 5 μM and 10 μM GSI-XXI administration using the same conditions.

### ALDH activity and CD133 staining

The ALDH activity was checked using ALDEFLUOR™ detection kit (StemCell Technology, 01700) following the kit protocol and data was acquired on FACS Caliber and analysis was carried out using CellQuest software. For CD133 staining was performed using CD133 (AC133) antibody (MACS Miltenyi Biotech) in FACS buffer for 15 min in dark at 4 °C. The cells were then washed twice with staining buffer and acquired on FACS Caliber, BD Biosciences.

### β-Galactosidase activity staining

Ten thousand cells were seeded in 12 well plates in triplicates and next day, AW13516 cells, vector control and overexpressing full length NOTCH1 were washed with 1X PBS and fixed with 0.5ml of fixative solution in the Abcam Senescence detection kit (Ab65351) for 10–15min at 25°C. Fixed cells then washed twice with 1X PBS and incubated for 8 hours with 0.5ml of staining solution containing 20mg/ml of X-gal. Stained cells were microscopically analyzed using Olympus IX-71. Images were analyzed using Image J and percentage β-Galactosidase positive cells were plotted.

### Survival and Statistical analysis

The relative impact of Notch pathway alterations on disease free survival (DFS) of TSCC patients was analyzed using Kaplan-Meier method [60] and was compared using the log-rank test for statistical significance. Data are expressed as mean ± standard deviation (SD) or standard error (SE). Significant differences between selected two groups were estimated using unpaired Student t-test using Graph Pad prism version 5. Statistical significance was set at p ≤ 0.05. Pearson correlation analysis and chi-square tests were performed in IBM SPSS statistics software version 21 for correlation analysis.

## SUPPLEMENTARY MATERIALS FIGURES AND TABLES


