# Supplementary material for: Notch pathway activation is essential for maintenance of stem-like cells in early tongue cancer
Source: Oncotarget. 2016 Jul 6;7(31):50437–49. doi: 10.18632/oncotarget.10419 (PMC5226594; doi:10.18632/oncotarget.10419)
Supplement: Supplementary file 1 [file oncotarget-07-50437-s001.pdf]

Notch pathway activation is essential for maintenance of stem-like cells in early tongue cancer

SUPPLEMENTARY FIGURES AND TABLES

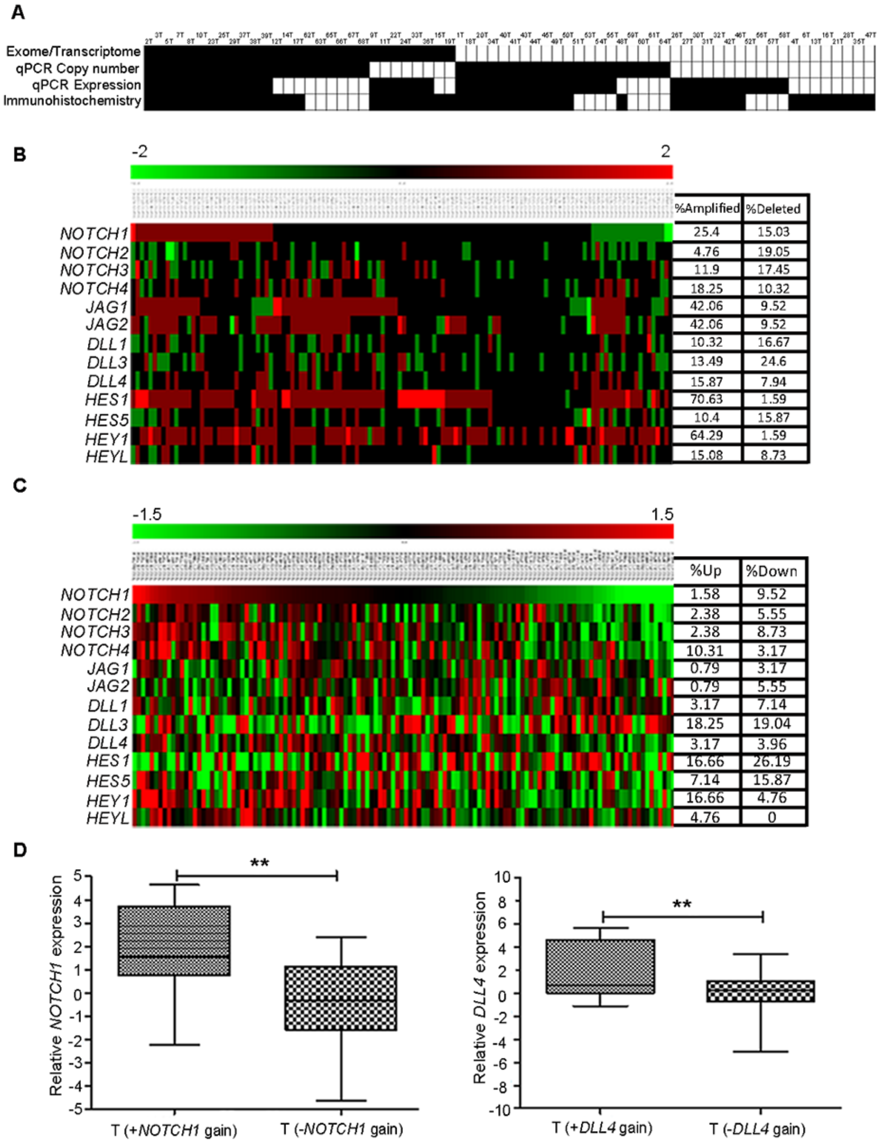

Supplementary Figure S1: Study overview and Notch pathway genes copy number and expression analysis in TCGA tongue tumors data. A. Study overview for Notch pathway genes in this study. Filled black box denotes sample was analyzed. **B.** The TCGA DNA copy number data analysis of tongue cancer patients (n=126) for Notch pathway genes. Data for Notch pathway genes (n=13) for tongue cancer patients has been retrieved. In the dataset gene-level copy number estimated values to -2,-1,0,1,2, representing homozygous deletion, single copy deletion, diploid normal copy, low-level copy number amplification, or high-level copy number amplification and heatmap were generated using MeV 4.9.0. Colors denotes: Red; high copy number, green; low copy number and black; diploid normal copy. **C.** The Expression data analysis of tongue cancer patients (n=126) for Notch pathway genes. Colors denotes: Red; higher expression, green; lower expression and black; no change. **D.** Box plot representation of *NOTCH1* and *DLL4* gene harboring DNA copy number gain in tongue tumor samples and their respective changes in gene expression was plotted. T, denotes Tumor and + and – denotes with and without DNA copy number gain or diploid. P value was calculated by Unpaired t-test, two sided considering P value ≤0.05 as threshold for statistical significance.

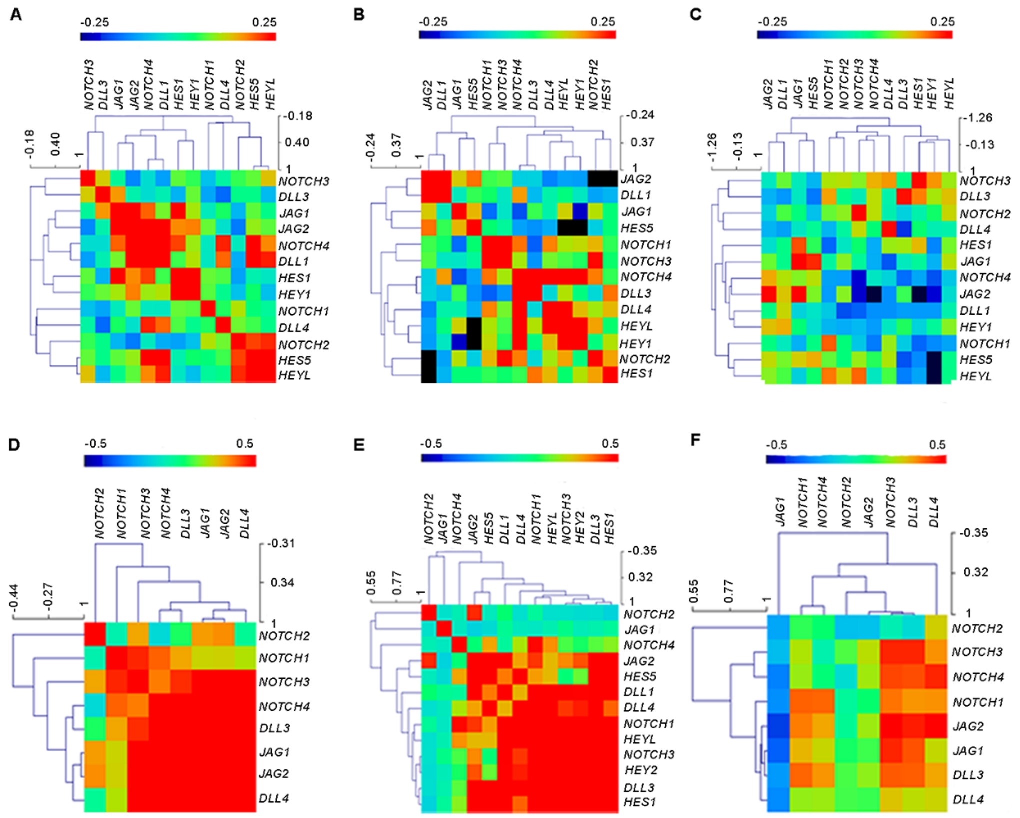

Supplementary Figure S2: Pearson Correlation analysis of DNA copy number and expression changes. Pearson Correlation analysis was performed in SPSS for DNA copy number and gene expression changes. Using Pearson correlation coefficient (R^2) obtained from SPSS analysis, the heatmap and clustering was performed in MeV software for three comparisons. **A.** and **D.** DNA copy number to DNA copy number, **B.** and **E.** Gene expression to gene expression and **C.** and **F.** DNA copy number to gene expression. A, B & C for TCGA cohort and D, E and F for our TSCC cohort.

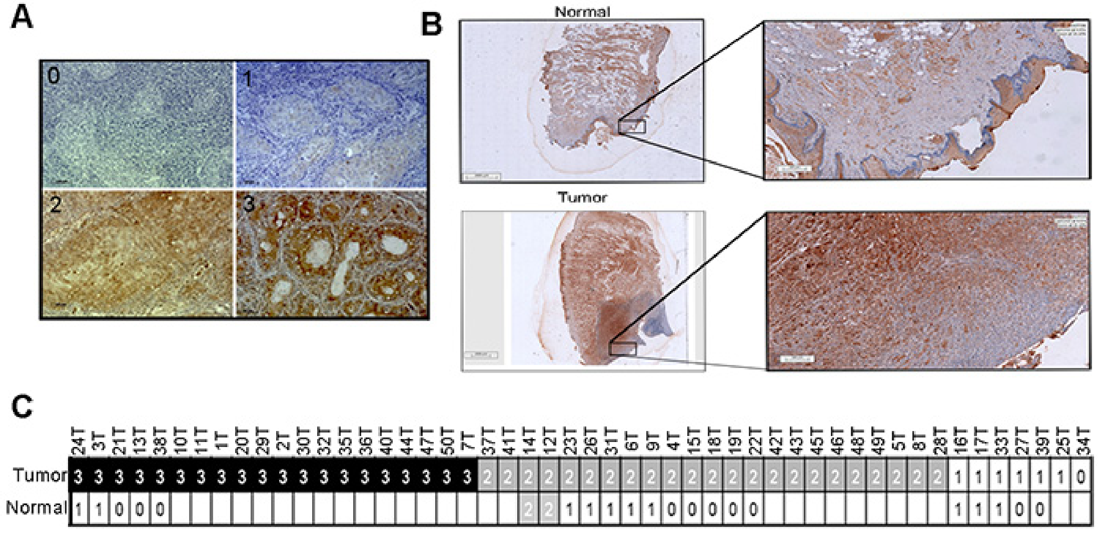

Supplementary Figure S3: Activated NOTCH1 immunohistochemistry in TSCC tumor samples. A. Representativeimages of different scoring pattern is shown: 0 or 1; no or weak, 2; moderate, 3; strong staining. **B.** Slide overview and zoom in image of representative TSCC normal and tumor tissue for activated NOTCH1 staining. **C.** Schematic representation of immunohistochemical scores of IHC slides. Immunostaining scores obtained from each sample is indicated.

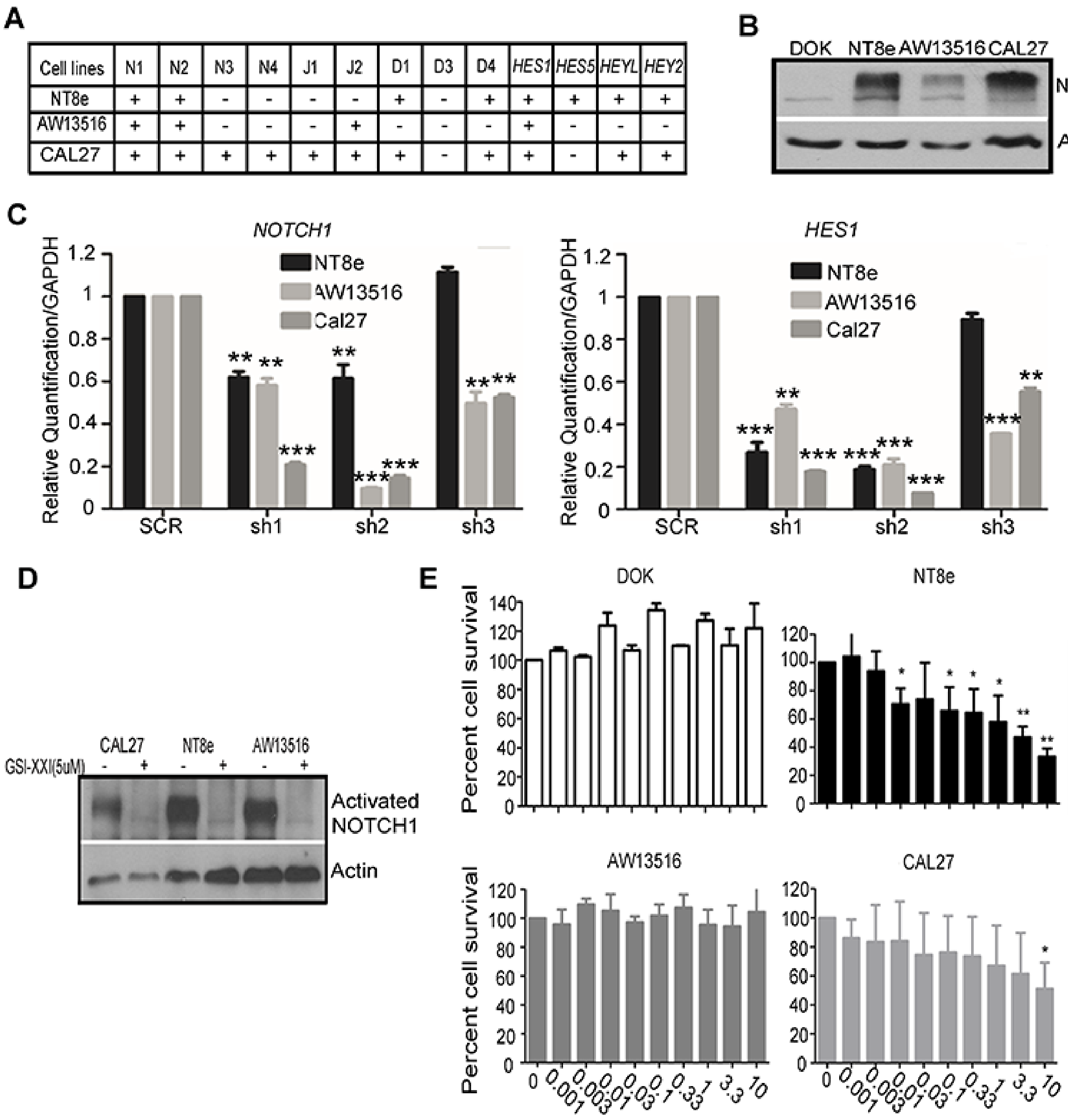

Supplementary Figure S4: Western blot and quantitative real time PCR analysis based analysis of Notch pathway and effect of GSI-XXI on HNSCC cells. A. qRT-PCR based analysis of *NOTCH1* transcript expression in NT8e, CAL27 and AW13516 cells. + & - denotes presence and absence of transcript, respectively. N1, J1 and D1 denotes *NOTCH1*, *JAG1*, and *DLL1* transcripts **B.** Western blot analysis of NOTCH1 expression in HNSCC cells. The NOTCH1 indicated in the upper panel. Lower panel denotes loading control, blotted for Actin. **C.** Quantitative real time PCR analysis based knockdown confirmation of *NOTCH1* and *HES1* in NT8e and AW13516 cells. **D.** Western blot analysis of activated Notch1 (NICD) after gamma secretase inhibitor (XXI) treatment post 48 hours and actin was used as loading control. **E.** Cell lines were treated with different concentration of GSI-XXI for 48 hours and MTT assay was performed. Percent cell survival for individual cell lines was calculated and plotted using GraphPad prism version 5. Unpaired Student-t-test, two sided was used for calculating P value. Data is shown as mean ± SE. *P-value* is denoted as, ** ;P < 0.001 versus untreated.; * denote P < 0.05 versus untreated.

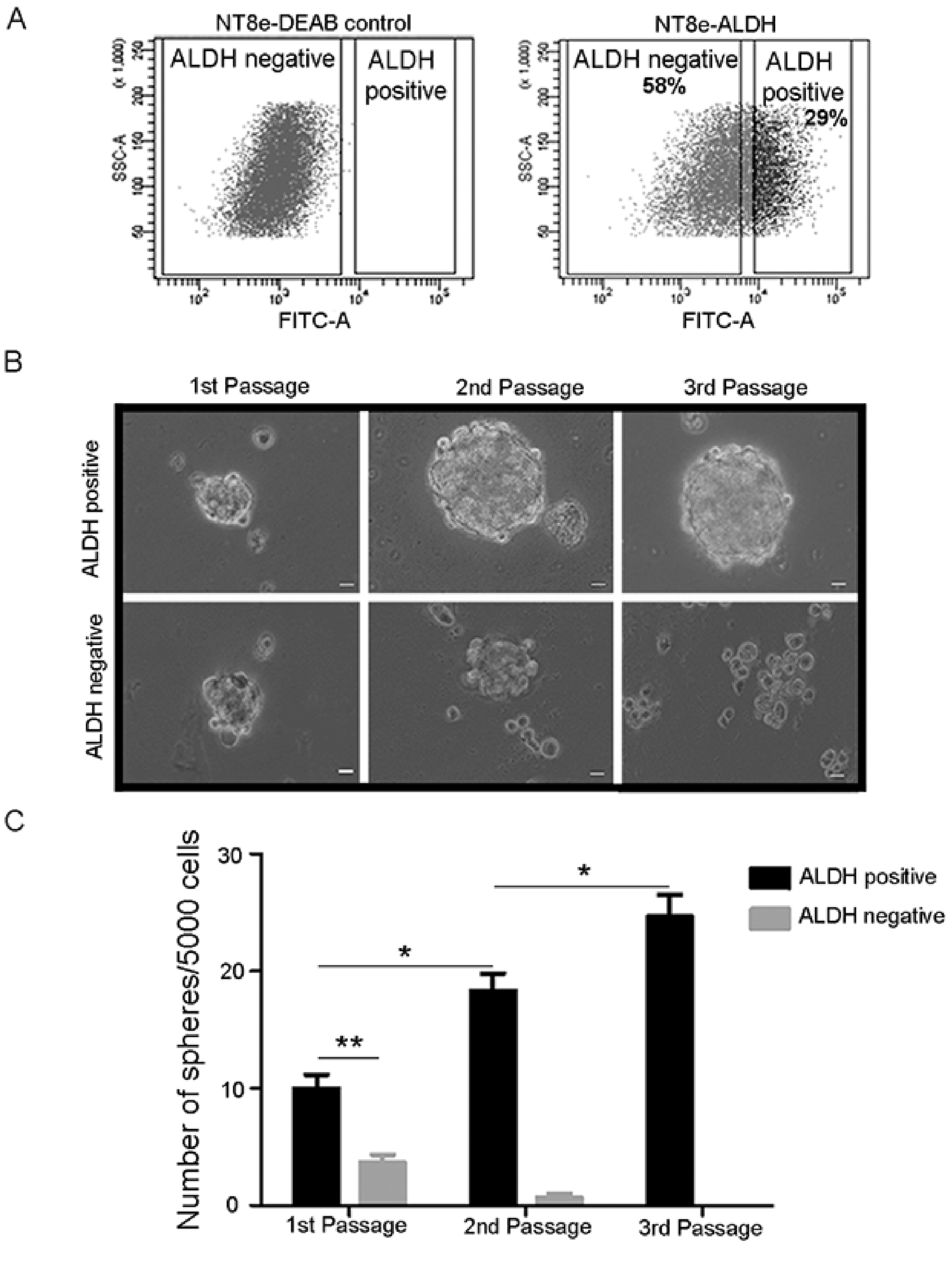

Supplementary Figure S5: Oralsphere formation assay of ALDH positive NT8e cells. A. Representative FACS analysis of ALDH activity. The ALDH positive and ALDH negative fractions in NT8e cells was determined by Aldefluor assay. Percentage ALDH positive and ALDH negative fractions are indicated at gated area. **B.** Representative images of the oralsphere formation of ALDH sorted fractions of NT8e cells taken at every passage at 20X magnifications. The sphere-forming efficiency was assessed for ALDH positive and ALDH negative fractions. Cells were grown under anchorage-independent cell culture media and sphere were counted on day 6 as indicated in material and methods section. **C.** Quantitative representation of sphere-forming ability of ALDH-sorted fractions of NT8e cells taken at every passage. Bar-graph reflects the means and SDs of three independent experiments. Results were considered statistically significant if, *P*- value <0.05 (* p<0.05, ** p <0.01).

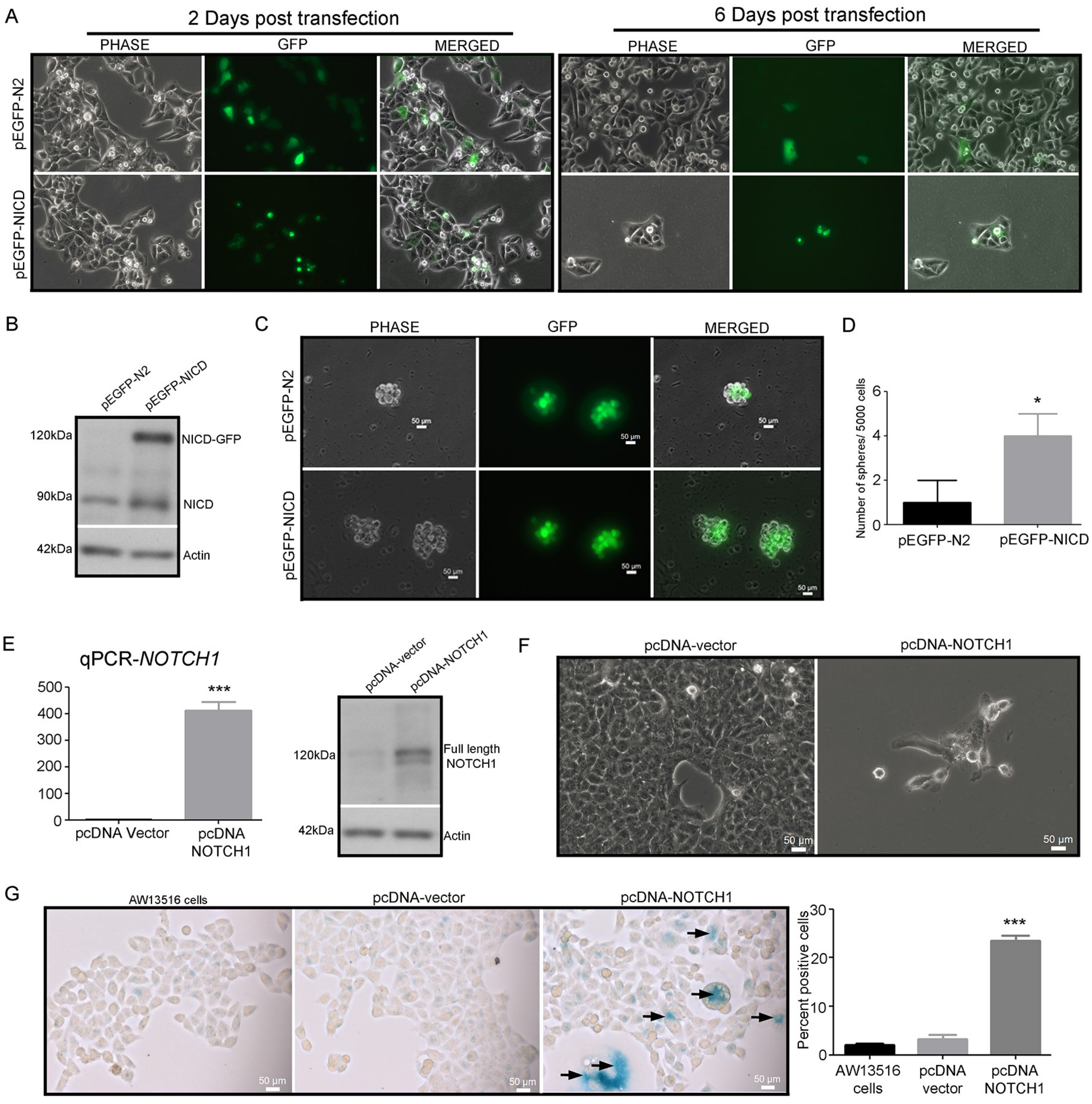

Supplementary Figure S6: Effect of NOTCH1 overexpression on AW13516 cells. A. Representative images of AW13516 cells transfected with vector control (pEGFP-N2) and NICD (pEGFP-NICD) and images were acquired at 20X magnification post 2 and 6 days of transfection. Cell growth arrest was observed in NICD-GFP transfected cells as compared to vector control. **B.** Western blot analysis of NICD-GFP expression in 293T cells post 48 hours of transfection. Endogenous NICD is shown at ~90 kDa and exogenous (NICD-GFP) at ~120 kDa. Actin was used as loading control. Overexpression was observed in NICD-GFP transfected cells. **C.** Representative images of oralsphere formation ability of AW13516 cells overexpressing NICD-GFP. AW13516 cells sorted using GFP were seeded for oralsphere assay for growing under anchorage-independent cell culture media and images were acquired post 6 days at 20X magnification. **D.** Quantitative bar graph representation of sphere-forming ability in AW13516 cells overexpressing NICD-GFP cells. **E.** Full length NOTCH1 overexpression confirmation in 293T cells. **Left panel:** Quantitative real-time PCR analysis of full length NOTCH1 transcript expression in 293T cells. **Right panel:** Western blot analysis of full length NOTCH1 overexpression in 293T cells post 48 hours of transfection. **F.** Representative images of AW13516 cells transfected with vector control (pcDNA-vector) and full-length *NOTCH1* (pcDNA-NOTCH1) and images were acquired at 20X magnification two days post neomycin selection. Cell growth arrest was observed in full-length *NOTCH1* transfected cells as compared to vector control. **G.** NOTCH1 induced senescence observed in AW13516 cells using β-Galactosidase activity staining assay. **Left panel:** Representative image of β-Galactosidase positive (indicated by black arrow, in blue color) AW13516 cells overexpressing full length NOTCH1 (day 15 post neomycin selection). **Right panel:** Quantitative bar graph representation of percentage β-Galactosidase positive cells in AW13516.Results were considered statistically significant if, *P*- value <0.05 (* $p<0.05$; *** $p<0.0001$).

A

ID	Survival (Months)	NOTCH1			*DLL4* copy number	Nodal Status
		Amplified	Overexpressed	IHC positive		
34T	30			1		
44T	17			■ 3	■	■
14T	22	■		■ 2	■	■
8T	23			■ 2	■	
40T	7	■	■	■ 3	■	■
3T	17		■	■ 3		■
7T	13			■ 3	■	■
9T	12		■	■ 2		■

B

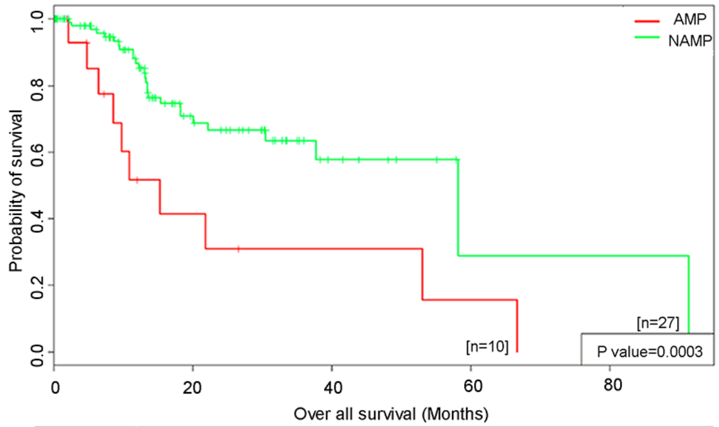

Status	*DLL4* Copy number (this study)						
	No of Samples	Observed (O)	Expected (E)	(O-E)^2/E	Chi-square value	Degree of freedom	P value
Amplified	19	6	3.61	1.57	2.9	1	0.0897
Not amplified	17	2	4.39	1.3			
	DLL4 Copy number (TCGA-tongue cancer)						
Status	N	Observed (O)	Expected (E)	(O-E)^2/E	Chi-square value	Degree of freedom	P value
Amplified	17	10	4.42	7.034	8.6	1	0.0033
Not amplified	107	27	32.58	0.955			

C

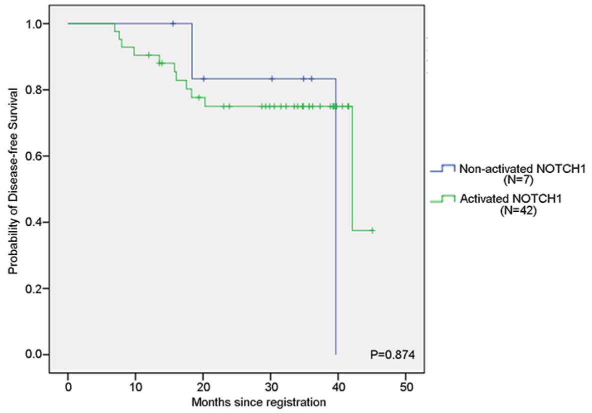

Supplementary Figure S7: Survival data of patient harboring NOTCH1 and *DLL4* alterations. A. Survival duration and clinical features representation of patients which met fatal event and their status for NOCH1 and *DLL4*. The black filled box denotes positive and while for negative. Immunohistochemistry staining score are denoted in number, 1; weak, 2; moderate, 3; strong staining of activated NOTCH1. **B.** Kaplan–Meier survival analysis of TSCC patients with or without *DLL4* amplification. Survival analysis of tongue cancer patients with and without *DLL4* amplification in TCGA- tongue cancer. AMP; amplified and NAMP; not amplified. Death of patients was taken as end point of analysis and P value ≤0.05 was considered as threshold for statistical significance. C. Disease-free survival analysis by immunohistochemistry defined NOTCH1 activation in tongue cancer patients. Patients were followed up and disease-free survival (DFS) analyzed by Kaplan–Meier survival analysis and survival difference was compared using log- rank test for statistical significance. There was no statistically significant difference in DFS between patients with activated vs non-activated NOTCH1 status; however the sample size is underpowered to detect significant difference. Numerically, the patients with activated NOTCH1 tumors has an inferior DFS as compared to those with NOTCH1 non-activated" tumors.

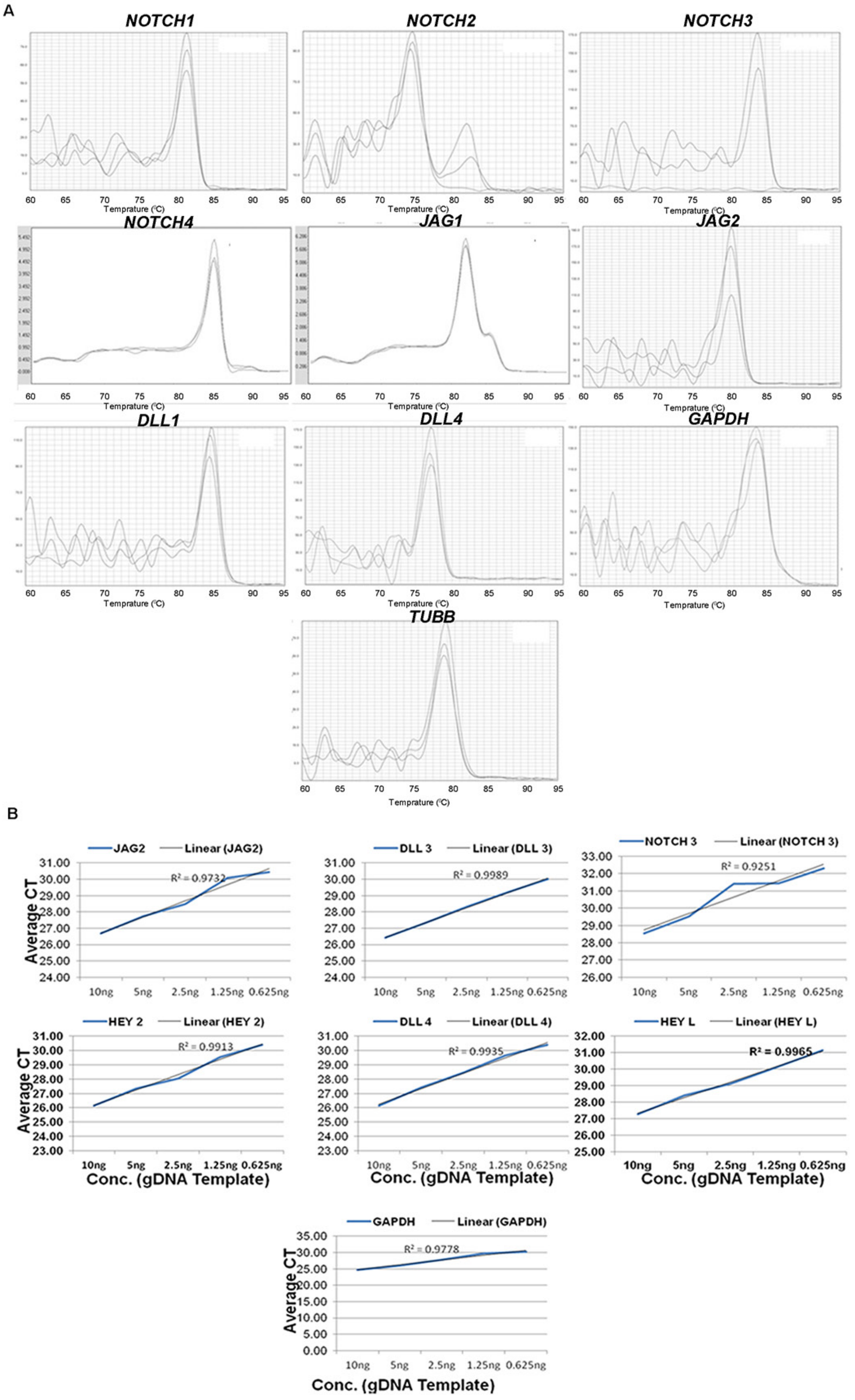

Supplementary Figure S8: Melt curve and amplification efficiency analysis of primers used for Copy number analysis. **A.** Melt curve analysis of Notch pathway genes. Distinct peak suggest that primers are amplifying single amplicon and less dimer formation. **B.** Amplification efficiency was performed with series of dilutions of genomic DNA.

Supplementary Table S1: List of Notch pathway gene (n=48) and mutations identified in the study

S.no.	Gene Name	Mutation	Number of samples mutated	Functional Class
1	*JAG1*	None	0	Ligand
2	*JAG2*	None	0	Ligand
3	*DLL1*	None	0	Ligand
4	*DLL3*	None	0	Ligand
5	*DLL4*	p.R100G	69T,9T	Ligand
6	*NOTCH1*	p.R780Q	70T	receptor
		p.D573A	8T	
		p.T859P	12T	
		p.A465T	38T	
		p.C554*	24T	
7	*NOTCH2*	None	0	receptor
8	*NOTCH3*	None	0	receptor
9	*NOTCH4*	p.LLLLL12fs	23T	receptor
10	*DVL1*	None	0	Negative regulator
11	*DVL2*	None	0	Negative regulator
12	*DVL3*	None	0	Negative regulator
13	*EP300*	None	0	Coactivator
14	*MAML1*	None	0	Coactivator
15	*MAML2*	None	0	Coactivator
16	*MAML3*	p.QQ508fs	25T	Coactivator
17	*SNW1*	None	0	Coactivator
18	*CREBBP*	None	0	Coactivator
19	*HDAC1*	None	0	Co-repressor
20	*HDAC2*	None	0	Co-repressor
21	*NCOR2*	None	0	Co-repressor
22	*NUMB*	None	0	NOTCH1 negative regulator
23	*NUMBL*	None	0	NOTCH1 negative regulator
24	*KAT2A*	None	0	Transcription Regulator
25	*KAT2B*	None	0	Transcription Regulator
26	*DTX1*	p.D122E	2T	NOTCH1 Positive regulator
27	*DTX2*	None	0	NOTCH1 Positive regulator
28	*DTX3*	None	0	NOTCH1 Positive regulator
29	*DTX3L*	None	0	NOTCH1 Positive regulator
30	*DTX4*	None	0	NOTCH1 Positive regulator
31	*LFNG*	None	0	Modifier (likely positive regulator)

(*Continued*)

S.no.	Gene Name	Mutation	Number of samples mutated	Functional Class
32	*MFNG*	None	0	Modifier (likely positive regulator)
33	*RFNG*	None	0	Modifier (likely positive regulator)
34	*PSEN1*	None	0	Secretase complex(receptor proteolysis)
35	*PSEN2*	None	0	Secretase complex(receptor proteolysis)
36	*PSENEN*	None	0	Secretase complex(receptor proteolysis)
37	*NCSTN*	None	0	Secretase complex(receptor proteolysis)
38	*APH1A*	None	0	Secretase complex(receptor proteolysis)
39	*ADAM17*	None	0	receptor proteolysis
40	*RBPJ*	p.M1R	70T	Transcription factor
41	*RBPJL*	None	0	Transcription factor
42	*CIR1*	None	0	Transcription factor
43	*HES1*	None	0	Downstream effector
44	*HES5*	None	0	Downstream effector
45	*HEY1*	None	0	Downstream effector
46	*PTCRA*	None	0	others
47	*HEYL*	None	0	Downstream effector
48	*HEY2*	None	0	Downstream effector

Supplementary Table S2: Details of correlation between clinicopathologic features of tongue cancer patients by IHC defined activated NOTCH1 status

Clinicopathologic features	Variable	N (% along column)	Activated NOTCH1 staining (N=49) N (% along row)		P- value*
			Strong and Moderate (N=42)	Weak (N=7)	
Age	<45 years	22 (45%)	18 (82%)	4 (18%)	0.68
	>45 years	27 (55%)	24 (89%)	3 (11%)	
Sex	Male	33 (67%)	30 (91%)	3 (9%)	0.19
	Female	16 (33%)	12 (75%)	4 (25%)	
AJCC Stage	I-II	19 (38%)	13 (68%)	6 (32%)	**0.01**
	III-IVA	30 (61%)	29 (97%)	1 (3%)	
Alcohol	No	35 (71%)	29 (83%)	6 (17%)	0.65
	Yes	14 (29%)	13 (93%)	1 (7%)	
Tobacoo	No	20 (41%)	16 (80%)	4 (20%)	0.42
	Yes	29 (59%)	26 (90%)	3 (10%)	

Highlighted P-value are statistically significant or marginal significance. * Fisher-exact test

Supplementary Table S3: The demographic and clinical characteristics of 68 tongue tumor samples used in the present study

Variable	Frequency (N=68)
Age, median (range)	42(23-76)
Sex	
Male	48(71%)
Female	20(29%)
Sub-site	
Oral tongue	100%
Tumor Stage	
T1	9(14%)
T2	55(86%)
Nodal Stage	
Node Negative	26(41%)
Node Positive	37(58%)
Habit	
Yes	55(81%)
No	13(19%)
Smoking	
Non-Smoker	47(69%)
Smoker	16(22%)
Alcohol	
Yes	43(74%)
No	15(26%)

Supplementary Table S4: Details of Primer sequences for Notch pathway gene used for DNA copy number (CNV) and expression (EXP). 5' and 3' denoted forward and reverse orientation of primer

Primer	Sequence	Primer	Sequence
NOTCH1 5'_CNV_EXP	GTGACTGCTCCCTCAACTTCAAT	*HES1* 5'_EXP	TCAACACGACACCGGATAAA
NOTCH1 3'_CNV_EXP	CTGTCACAGTGGCCGTCACT	*HES1* 3'_EXP	TCAGCTGGCTCAGACTTTCA
NOTCH2 5'_CNV_EXP	GGCATTAATCGCTACAGTTGT GTCT	*HES5* 5'_EXP	CTCAGCCCCAAAGAGAAAAA
NOTCH2 3'_CNV_EXP	GGAGGCACACTCATCAATGTCA	*HES5* 3'_EXP	GACAGCCATCTCCAGGATGT
NOTCH3 5'_CNV_EXP	TGATCGGCTCGGTAGTAATGC	*HEY2* 5'_EXP	TCGCCTCTCCACAACTTCAGA
NOTCH3 3'_CNV_EXP	GACAACGCTCCCAGGTAGTCA	*HEY2* 3'_EXP	GAATCCGCATGGGCAAAC
TUBULIN 5'_CNV_EXP	CTACAATGCCACCCTCTCCG	*HEYL* 5'_EXP	GGCTGCTTACGTGGCTGTT
TUBULIN 3'_CNV_EXP	CTTCAGAGTGCGGAAGCAGA	*HEYL* 3'_EXP	GACCCAGGAGTGGTAGAGCAT
DLL4 5'_CNV_EXP	GTCTCCACGCCGGTATTGG	*JAG1* 5'_EXP	GCCGAGGTCCTATACGTTGC
DLL4 3'_CNV_EXP	CAGGTGAAATTGAAGGGCAGT	*JAG1* 3'_EXP	CCGAGTGAGAAGCCTTTTCAA
JAG2 5'_CNV_EXP	GGCACTCGCTGTATGAAAGGA	*GAPDH* 5'_EXP	AATCCCATCACCATCTTCCA
JAG2 3'_CNV_EXP	GCACAACCTCTGGTAACAAACG	*GAPDH* 3'_EXP	TGGACTCCACGACGTACTCA
DLL3 5'_CNV_EXP	CCCTACCCTTCCTCGATTCTG	*NOTCH4* 5'_CNV	AAGGTACCCCAGGTGTCAGT
DLL3 3'_CNV_EXP	GAACTGAAAATGGGCTTAAA ACCTT	*NOTCH4* 3'_CNV	TTCAACCAGGTCTTCCACCG
NOTCH4 5'_EXP	AACTCCTCCCCAGGAATCTG	*JAG1* 5'_CNV	TGACCAAGCTCTGCTCAAGG
NOTCH4 3'_EXP	CCTCCATCCAGCAGAGGTT	*JAG1* 3'_CNV	GCAGACAAGTTGACGAGGGA
DLL1 5'_EXP	TGCAACCCTGGCTGGAAA	*GAPDH* 5'_CNV	GAGGCTCCCACCTTTCTCATC
DLL1 3'_EXP	AATCCATGCTGCTCATCACATC	*GAPDH* 3'_CNV	ATTATGGGAAAGCCAGTCCCC

copy number (CNV) and expression (EXP). 5' and 3' denoted forward and reverse orientation of primer.

Supplementary Table S5: STR profile of all cell lines used in the study

Cell line	STR marker's									
	**TH01**	**D21S11**	**D5S818**	**D13S317**	**D7S820**	**D16S539**	**CSF1PO**	**AMEL**	**vWA**	**TPOX**
NT8e	8	31.2,35.2	12,13	12	12	8	12	X	17,18	9,11
AW13516	8	28	9,11	12	10,12	12	11,12	X	14,17	11
CAL27	6,9.3	28,29	11,12	10,11	10	11,12	10,12	X	14,17	8
DOK	6,8	29,31	12	11,13	11,13	9,12	12	X,Y	14,18	10
